# T1R3 Anchors ACE2 at the Pulmonary Endothelial Cell Surface to Attenuate Vascular Injury Following SARS‐CoV‐2 Spike 1 Protein Exposure In Vitro

**DOI:** 10.1111/jcmm.71339

**Published:** 2026-08-26

**Authors:** Zsuzsanna Kertesz, Lewis Spurrier‐Best, Havovi Chichger

**Affiliations:** ^1^ Centre for Biomedical Research and Health Innovation Anglia Ruskin University Cambridge UK

**Keywords:** ACE2, COVID‐19, endothelium, lung, SARS‐CoV‐2, T1R3

## Abstract

The development of acute lung injury in COVID‐19 patients is a major cause of lethality. Activation of the pulmonary vasculature, with inflammation, oxidative stress and barrier disruption, is initiated by the SARS‐CoV‐2 spike protein (Sp1) at the endothelial cell surface. We previously identified the presence of a novel G‐Protein Coupled Receptor (GPCR) in the lung microvasculature, T1R3. The T1R3 agonist (T1R3‐A) protects the endothelium from endotoxin‐induced lung injury therefore we sought to investigate the effect of T1R3‐A on Sp1‐induced damage to the pulmonary endothelium. Sp1 induced significant disruption to the endothelium with elevated gap formation, monolayer leak and ROS accumulation. This damage was attenuated following exposure of the lung microvasculature to T1R3‐A. We further demonstrated that this agonist promotes ACE2 expression at the endothelial cell surface. Using immunoprecipitation studies, we demonstrated that T1R3‐A increases ACE2:T1R3 binding which may be a mechanism for protecting against Sp1‐induced endothelial cell injury. Our findings highlight the protective effect of T1R3 activation in the pulmonary endothelium and that there is a close link between T1R3 and ACE2. We propose that T1R3 activation protects the endothelium by preventing Sp1‐induced internalisation of ACE2, thereby blocking downstream ACE2 signalling leading to pulmonary vascular injury.

## 1 Introduction

Acute respiratory distress syndrome (ARDS) is a significant contributor to intensive care unit, responsible for approximately 10% of all admissions and up to 23% of all mechanically ventilated patients [1]. ARDS is associated with 43% overall mortality, broken down into 45% mortality for severe ARDS patients and 27% mortality for patients with mild ARDS [2, 3]. A key hallmark of ARDS is inflammation and increased endothelial permeability in the lung microvasculature with a subsequent build‐up of fluid in the alveolar space [4]. Eventually, this fluid accumulation impairs gas exchange resulting in hypoxemia and a need for mechanical ventilation for patients, although this treatment is associated with further lung injury for some patients [5]. As a result, pharmacological intervention for patients with ARDS has been sought; however, despite the range of studies performed in the field, there are surprisingly few pharmacological treatment options for these patients. The main causes of ARDS are sepsis, trauma, pneumonia and gastric aspiration, and more recently, COVID‐19 was identified to contribute to 75% of ARDS patients in ICU and 90% mortality in these patients [6]. Although the COVID‐19 pandemic has now passed, there is significant interest on the pathological effect of SARS‐CoV‐2 on the lung and the resulting link with ARDS development to help understand molecular mechanisms underpinning virus‐induced lung injury.

A key feature of ARDS in patients with COVID‐19 is severe hypoxaemia despite relatively compliant lungs with well‐preserved lung mechanics [7]. This is suggestive of a loss of lung perfusion associated with pulmonary endothelial cell dysfunction termed endothelialitis. Indeed, severe endothelial injury is evident in patients with clinical features including perivascular T‐cell infiltration, widespread thrombosis and microangiopathy [7, 8]. Treatment with either the SARS‐CoV‐2 virus or sera from patients with COVID‐19 significantly increases markers of endothelial cell activation and injury accompanied by breakdown of the endothelial monolayer [9]. The presence of the cellular machinery which recognises and internalises the SARS‐CoV‐2 virus in the endothelium, angiotensin‐converting enzyme‐2 (ACE‐2), has been shown to mediate this virus‐induced endothelial cell damage which is the basis of lung injury in patients [10, 11]. Interestingly, the S1 spike protein from SARS‐CoV‐2 (Sp1) has also been shown to cause endothelial cell activation, excessive inflammation and breakdown of the pulmonary endothelial monolayer [12]. Sp1 protein contains the receptor‐binding domain (RBD) which binds to ACE2 on the endothelial cell membrane to allow SARS‐CoV‐2 entry into the cell [13]. Sp1 causes activation of the TGF‐β pathway to elevate expression of GAGs and integrins, along with other pro‐inflammatory molecules such as IL‐6, MCP‐1 and PAI‐1 [14, 15, 16]. This pro‐inflammatory cascade is associated with vascular leak, demonstrating that viral infection is not the primary cause of vascular damage. Whilst these studies demonstrate a clear link between Sp1 and endothelial cell injury seen in patients with SARS‐CoV‐2 infection, there is still a lack of understanding of how to block this signalling and improve outcomes for patients with ARDS induced by COVID‐19 or other viruses.

We have previously identified the expression of novel G‐protein coupled receptors (GPCRs), T1R and T2R, in the pulmonary endothelium which are linked to the taste sensing pathway [17, 18]. Agonists for the sweet taste receptor, T1R3 significantly attenuate pulmonary endothelial barrier disruption caused by endotoxin‐ or bacteria‐induced models of ARDS in vitro and in vivo respectively [18]. The T1R3 agonist (T1R3‐A), sucralose (Suc) attenuates LPS‐induced Src, PAK and MLC2 phosphorylation in lung microvascular endothelial cells to maintain VE‐cadherin expression at the endothelial cell surface and form a tighter endothelial monolayer [18], however studies have focused on endothelial injury caused by LPS. There is an intriguing link between taste sensing and SARS‐CoV‐2 with co‐localisation of T1R3 and key signalling molecules in the renin‐angiotensin system in oral taste tissue [19]. In silico studies indicate that the RBD of Sp1 interacts with high affinity to bitter taste receptors such as T2R4 and T2R10 whereas immunoprecipitation studies demonstrate ACE2‐T1R3 binding [20, 21]. This literature suggests that taste receptors are closely linked to Sp1‐ACE2 signalling; however there are no studies which investigate the crosstalk between T1R and ACE2 or how this may affect the pulmonary endothelium. Therefore, in the present study, we sought to evaluate the effect of a T1R3 agonist on Sp1‐induced injury to the lung microvasculature in vitro. We aimed to identify a novel molecule which regulates Sp1‐ACE2 signalling in the endothelial cell with the hope of shedding light on a new potential therapeutic approach to treating lung injury in settings of viral infection.

## 2 Materials and Methods

### 2.1 Materials

Human pulmonary microvascular endothelial cells (HPMVEC) and endothelial cell growth medium MV and Supplement pack were obtained from PromoCell. The antibiotics (1% penicillin and streptomycin), trypsin/EDTA and porcine gelatine used in in vitro studies were from Sigma. Cytokine levels, IL‐6 (D6050), IL‐8 (D8000C), IL‐1β (DLB50), IL‐18 (DL180) and TGF‐β (DB100C) were assessed using Quantikine ELISA kits purchased from R&D Systems (Abingdon, UK). siRNA transfection reagents, SMARTpool siGENOME T1R3 and non‐specific scrambled siRNA duplexes and Dharmafect reagent, were purchased from Dharmacon (Cambridge, UK). Recombinant SARS‐CoV‐2 spike protein S1 subunit (Sp1; 230–01101‐500) was obtained from Cambridge Bioscience (Cambridge, UK). VE‐cadherin (MABT129) and pan‐cadherin (C1821) were purchased from Merck Millipore (Watford, UK) and ACE2 (Ab108252) and PECAM‐1 (Ab9498) antibodies were purchased from Abcam (Cambridge, UK). The actin (C‐4) and T1R3 (sc‐398996) were purchased from Santa Cruz Biotechnology (Middlesex, UK). TMPRSS2 (PA5‐116054) and phosphorylated VE‐cadherin (Y658, 44‐1144G), MEM‐PER Plus Membrane Protein kit, and human‐specific primers for *T1R3*, *ACE2* and *GADPH*, designed based on previously published studies [17, 18, 22], were purchased from Thermo Fisher Scientific (Paisley UK). ICAM‐1 (MEM‐111) antibody was purchased from Novus Biologicals (Abingdon, UK). All other materials, including RIPA buffer and MTT, were obtained from Merck Life Science (Dorset, UK).

### 2.2 Cell Culture and Treatments

HPMVEC were cultured on gelatine‐coated tissue culture ware between passage 2–6 in endothelial cell growth medium supplemented with the bullet kit, antibiotics (1%) and foetal calf serum (5%). HPMVEC were serum‐starved (1% FCS) for 24 h prior to treatment. Sp1 peptide was prepared in media and used to treat HPMVEC at a range of concentrations from 1 to 100 nM for up to 24 h. T1R3‐agonist (T1R3‐A), sucralose, dynamin inhibitor, Dynasore and lactisole were used at concentrations of 100, 80 and 3 μM as previously published [18, 23, 24]. Endothelial cells were transiently transfected with siRNA specific to human *T1R3* (NM_152228) or a scrambled, non‐specific control duplex, using both at 300 nM concentrations, for 48 h, as per the manufacturer's guidelines. siRNA knockdown efficiency was demonstrated by Western blot and RT‐PCR with a specific anti‐human T1R3 antibody and specific *T1R3* primers, respectively.

### 2.3 mRNA Expression Analysis

Expression of *T1R3* and *ACE2* mRNA was measured using RT‐PCR with previously published human‐specific primers [17, 18, 22]. Total RNA was extracted from endothelial cells as previously described [18] and transcripts were measured using PCR primers. GAPDH was used as the housekeeping gene and relative gene expression levels were analysed using the ΔCt method where ΔCt = Ct_ACE2/T1R3_ = Ct_GAPDH_ and normalised to vehicle treatment.

### 2.4 Whole Cell ELISA

Cell surface expression of VE‐cadherin, ICAM‐1, ACE2 and TMPRSS2 was assessed using whole‐cell ELISA, as previously described [18], with antibodies specific to the extracellular region of each protein. Whole‐cell ELISA was performed on HPMVEC exposed to Sp1 and T1R3‐A, or following siRNA transfection, that were fixed with 1% paraformaldehyde (15 min, 4°C). Cells were then rinsed (PBS), blocked and incubated with 15 μg/mL monoclonal antibody. Incubation with antibody specific to human VE‐cadherin was followed by incubation with 488‐fluorescent‐conjugated secondary antibody (0.2 μg/mL) and measured at 485 nm excitation and 552 nm emission with a fluorescent plate reader (FLUOStar, BMG LabTech). Incubation with antibodies specific to extracellular regions of human ICAM‐1, T1R3, ACE2 and TMPRSS2 was followed by HRP‐conjugated secondary antibody incubation (0.2 μg/mL) and 3,3′,5,5′‐tetramethylbenzidine (TMB) substrate. Absorbance was assessed at 450 nm using a microplate reader (FLUOStar, BMG LabTech).

### 2.5 Immunoprecipitation, Membrane Preparation and Western Blotting

Membrane preparations were made using the Mem‐PERTM Plus Membrane protein extraction kit. Manufacturer's guidelines for ‘adhesion mammalian cells’ was followed using treated HPMVEC. Whole cell lysate was prepared by scraping cells, using RIPA buffer, on ice followed by centrifugation. For immunoprecipitation studies, 500 μg lysate was immunoprecipitated with ACE2 antibody overnight at 4°C. Immunocomplexes were incubated with protein G agarose beads for 4 h, rinsed and centrifuged. Whole cell lysate (50 μg), membrane pellet preparations (20 μg) or bead complexes were resuspended in Laemmli buffer and samples (50 μg) were analysed with Western blotting using 10% SDS‐PAGE and primary antibodies, used at 1 μg/mL, specific to ACE2, TMPRSS2 and pan‐cadherin. Secondary antibodies were used at 0.2 μg/mL with densitometry analysis performed using ImageJ software (Version 1.54j) and data was normalised to pan‐cadherin.

### 2.6 Endothelial Cell Viability, Oxidative Stress and Cytokine Measurements

For viability assays, HPMVEC were grown to 60% confluence prior to Sp1 treatment, or media vehicle, for 24 h. Cells were then incubated with MTT reagent (3‐(4,5‐Dimethylthiazol‐2‐yl)‐2,5‐Diphenyltetrazolium Bromide) for 2 h, both at 37°C. Following solubilisation with sodium dodecyl sulphate (SDS) overnight, absorbance was then assessed at 450 nm using a microplate reader. Cell viability changes were confirmed by performing a cell count (haemocytometer) on HPMVEC following 24 h treatment with Sp1 or media vehicle.

For oxidative stress accumulation assays, cells were cultured in black‐walled, clear‐bottomed 96‐well plates and pre‐exposed to 2′,7′–dichlorofluorescin diacetate (DCFDA) at 10 μM for 30 min in the dark. HPMVEC were rinsed twice in PBS followed by treatment with Sp1, in the presence and absence of T1R3‐A (100 μM) for 1 h. Fluorescence excitation/emission was measured using a fluorescent microplate reader.

Cytokine levels were assessed using Quantikine ELISA kits for IL‐6, IL‐8, IL‐1β and TGF‐β, following the manufacturer's guidelines. Absorbance was assessed after stop solution was added at 450 nm, correction to 540 nm, using a microplate reader.

### 2.7 Monolayer Permeability and Gap Formation Assays

Permeability of the endothelial monolayer was assessed using the fluorescein isothiocyanate (FITC)‐dextran assay, as previously described [17]. HPMVEC were seeded onto Transwell inserts in a 24‐well plate and cultured for 24 h followed by exposure to Sp1 and T1R3‐A or siRNA transfection for a further 24 h. At this time point, either trans‐endothelial electrical resistance (TEER) or FITC‐dextran permeability was measured. TEER was assessed using an Epithelial Volt/Ohm Meter Three (EVOM3), with the short probe placed in the apical chamber and the other longer probe in the corresponding basolateral well. A blank well containing only media (no cells) was used for background correction. Permeability was assessed using FITC‐conjugated 4 kD dextran (1 mg/mL) which was added to the upper chamber of the Transwell insert. Media was then collected from the lower chamber of the Transwell insert (100 μL) at 180 s and measured for FITC concentration at excitation of 485 nm and emission of 535 using a fluorescent plate reader. Permeability in relative fluorescence units (r.f.u.) was used to calculate % permeability as the fluorescence in the lower chamber divided by that in the upper chamber and multiplied by 100 and normalised to vehicle treatment.

Gap formation in the endothelial monolayer was measured using the XPert assay, as previously described [25]. HPMVEC were plated onto bioinylated gelatin‐coated plates and treated with Sp1 and T1R3‐A or siRNA transfection for 24 h, or untreated cell control. FITC‐avidin solution was added to the culture media for 3 min and unbound avidin was rinsed with PBS and quantified by excitation of 485 nm and emission of 535 using a fluorescent plate reader.

### 2.8 Data Analysis and Statistics

All data sets were statistically analysed using GraphPad Prism (Version 7.05). Analysis was performed using either a one‐way or two‐way ANOVA with Dunnett's Multiple comparisons post hoc test where relevant. Statistical significance is considered where *p* value is less than 0.05. Data is presented as mean ± standard error of the mean (SEM) unless otherwise stated and sample size (*n* number) is included in the figure legend for each study.

## 3 Results

### 3.1 Sp1 Peptide Significantly Disrupts Pulmonary Endothelial Barrier Function With Increased Oxidative Stress and Inflammation

**FIGURE 1:**
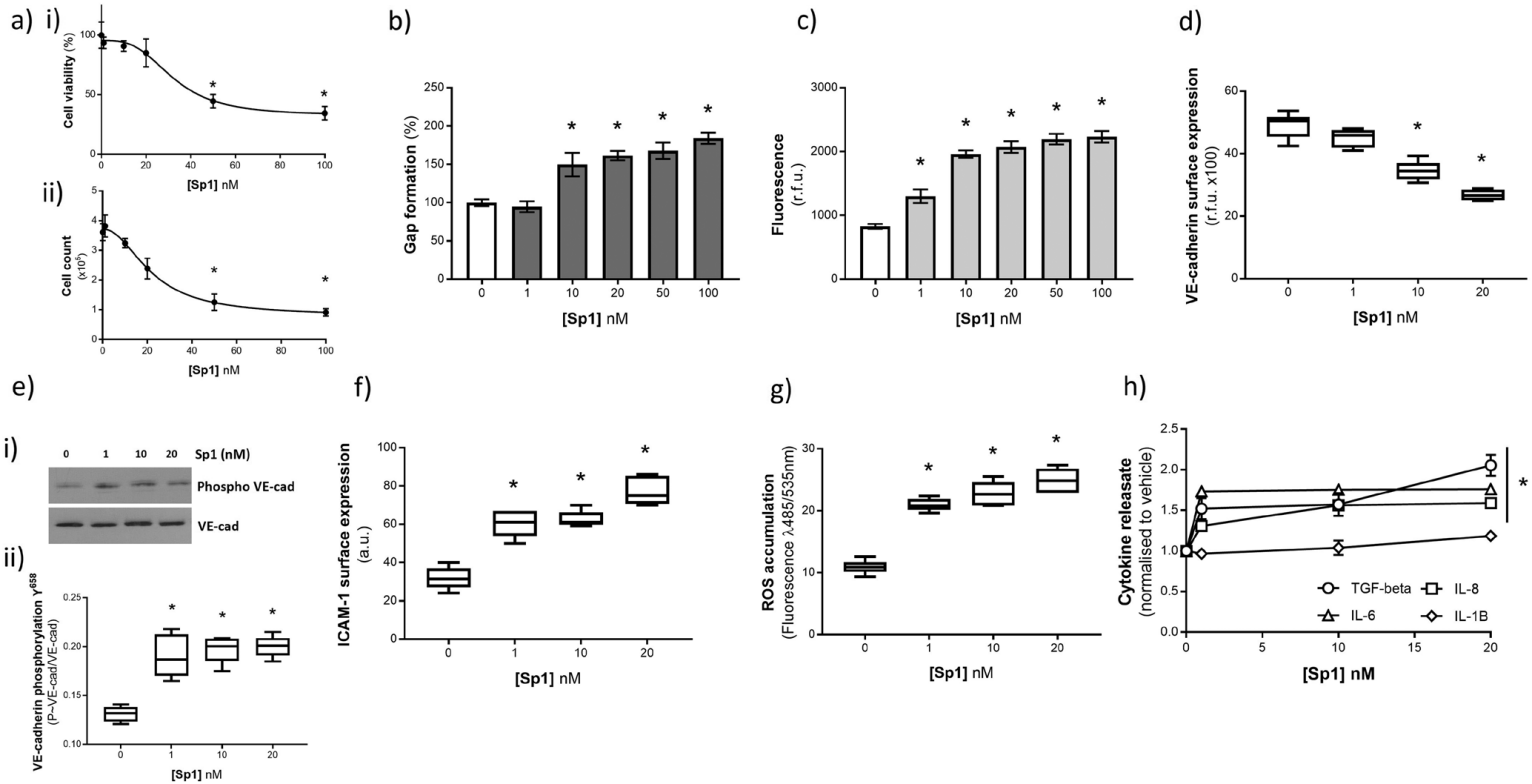
Sp1 peptide significantly disrupts pulmonary endothelial barrier function with increased oxidative stress and inflammation. HLMVEC were exposed to Sp1 at concentrations from 0 to 100 nM for 24 h or media control. Cell viability (a), gap formation (b) and monolayer permeability (c) were measured using MTT, XPert and FITC‐monolayer flux assay respectively. VE‐cadherin phosphorylation (d) was measured with Western blotting with representative blot (i) and quantification (ii). VE‐cadherin (e) and ICAM‐1 (f) endothelial cell surface expression was assessed with whole‐cell ELISA. ROS accumulation (g) and cytokine release (h) were measured using DCFDA and Quantikine kits. Data are presented as mean ± SEM. *n* = 6. *p < 0.05 versus vehicle‐treated (0 μM) cells.

To investigate the effect of recombinant SARS‐CoV‐2 spike protein S1 subunit (Sp1) on the pulmonary endothelium in vitro, we first sought to assess the concentration of Sp1 which caused HPMVEC toxicity. Current studies in the field focus on Sp1 concentrations from 8 to 266 nM which corresponds to circulating levels detected in critically ill COVID‐19 patients [26, 27]. In the current study, we lowered the range to more closely mimic COVID‐19 sufferers outside of intensive care units. Cell viability was studied at concentrations ranging from 1 to 100 nM. Our findings demonstrated a significant loss of microvascular endothelial cell viability and cell number with exposure to Sp1 at 50 and 100 nM (Figure 1a(i,ii)) with an IC50 of 40.7 ± 8.5 nM (mean ± SEM). Interestingly, pulmonary endothelial barrier integrity, measured as gap formation (Figure 1b) and flux of FITC‐dextran across the monolayer (Figure 1c), showed significant breakdown following exposure to Sp1 at concentrations of 10 nM and higher. At concentrations higher than 20 nM, it is likely that barrier disruption is due to endothelial cell death (Figure 1a); therefore, further studies focused on Sp1 concentration up to 20 nM. Cell surface expression of the vascular endothelial adherens junction protein, VE‐cadherin, was significantly downregulated at both 10 and 20 nM Sp1 exposure (Figure 1d) coupled with a significant increase in phosphorylation of VE‐cadherin at Y658 (Figure 1e). There was also a significant increase in ICAM‐1 expression (Figure 1f) and elevated ROS accumulation (Figure 1g) within the endothelial cells at Sp1 concentration of 1 nM and higher. This was accompanied with a significantly increase in release of IL‐8, IL‐6 and TGF‐β, but not IL‐1β, from pulmonary endothelial cells following exposure to Sp1 at concentrations of 1 nM and higher (Figure 1h). Taken together, these findings demonstrate that Sp1 causes significant breakdown of the pulmonary endothelial barrier in vitro and confirm a pro‐inflammatory and oxidative stress status of the cells as could be expected for a model of SARS‐CoV‐2 infection.

### 3.2 A T1R3 Agonist (T1R3‐A) Significantly Attenuates Sp1‐Induced Barrier Disruption and Oxidative Stress, but Not Inflammation, in the Pulmonary Endothelium In Vitro

**FIGURE 2:**
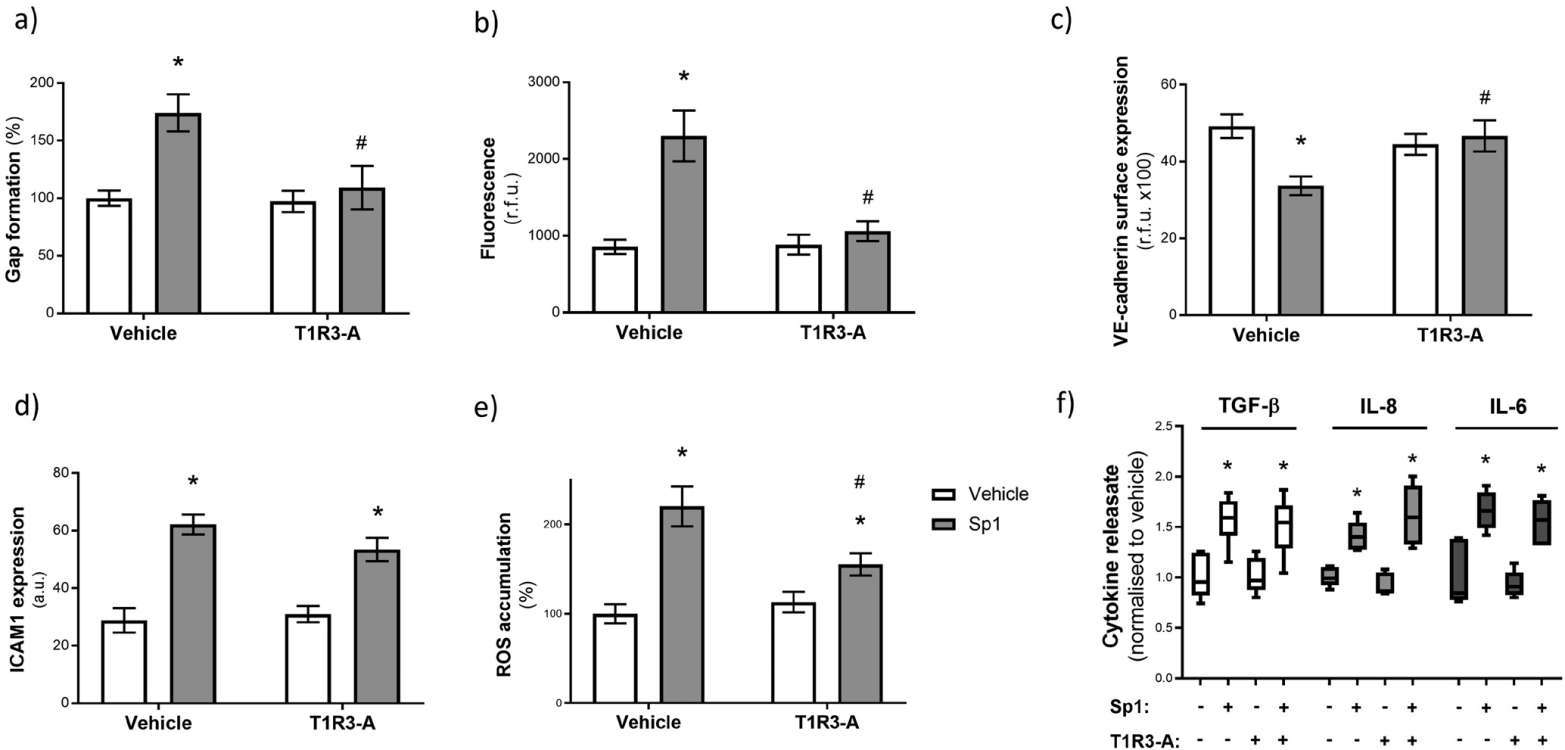
The T1R3 agonist, T1R3‐A, significantly attenuates Sp1‐induced barrier disruption and oxidative stress, but not inflammation, in the pulmonary endothelium in vitro. HLMVEC were treated with Sp1 (10 nM) or vehicle (media) with T1R3‐A (sucralose, 100 μM) or vehicle for sucralose (H_2_O) for 24 h. Gap formation (a) and monolayer permeability (b) were assessed using the XPert and FITC‐flux assay respectively. VE‐cadherin (c) and ICAM‐1 (d) surface expression was measured using whole‐cell ELISA, whilst ROS accumulation (e) and cytokine release of TGF‐β, IL‐6 and IL‐8 (f) was analysed with DCFDA and Quantikine kits, respectively. Data are presented as mean ± SEM. *n* = 6–7. *p < 0.05 versus vehicle for Sp1, ^#^ *p* < 0.05 versus vehicle for T1R3‐A.

Our previous findings have demonstrated a protective effect of a T1R3 agonist (T1R3‐A) in reducing pulmonary vascular leak in vitro and in vivo bacterial models of ARDS [18]. We next sought to understand whether T1R3‐A protected the pulmonary endothelium against Sp1‐mediated barrier disruption, oxidative stress and inflammatory cytokine release. Co‐exposure of T1R3‐A with Sp1 (10 nM) significantly attenuated gap formation (Figure 2a) and FITC‐dextran flux across the monolayer (Figure 2b) to baseline levels. This was accompanied by attenuation of Sp1‐induced reduction of cell surface VE‐cadherin expression (Figure 2c). Interestingly, the significant increase of ICAM‐1 surface expression (Figure 2d) and cytokine release (IL‐8, IL‐6 and TGF‐β; Figure 2f), which was induced by Sp1, was not affected by T1R3‐A. Whilst Sp1‐mediated ROS accumulation was significantly attenuated by T1R3‐A exposure, it was still not returned to baseline levels (Figure 2e).

These findings indicate a barrier‐protective role for T1R3‐A in reducing Sp1‐mediated damage to the pulmonary endothelium in vitro. Interestingly, this protective effect may be linked to oxidative stress but not inflammation, suggesting a specific role for T1R3‐A in Sp1‐induced signalling.

### 3.3 T1R3‐A Attenuates Sp1‐Induced Internalisation of ACE2 at the Pulmonary Endothelial Cell Surface Through Intracellular Trafficking

**FIGURE 3:**
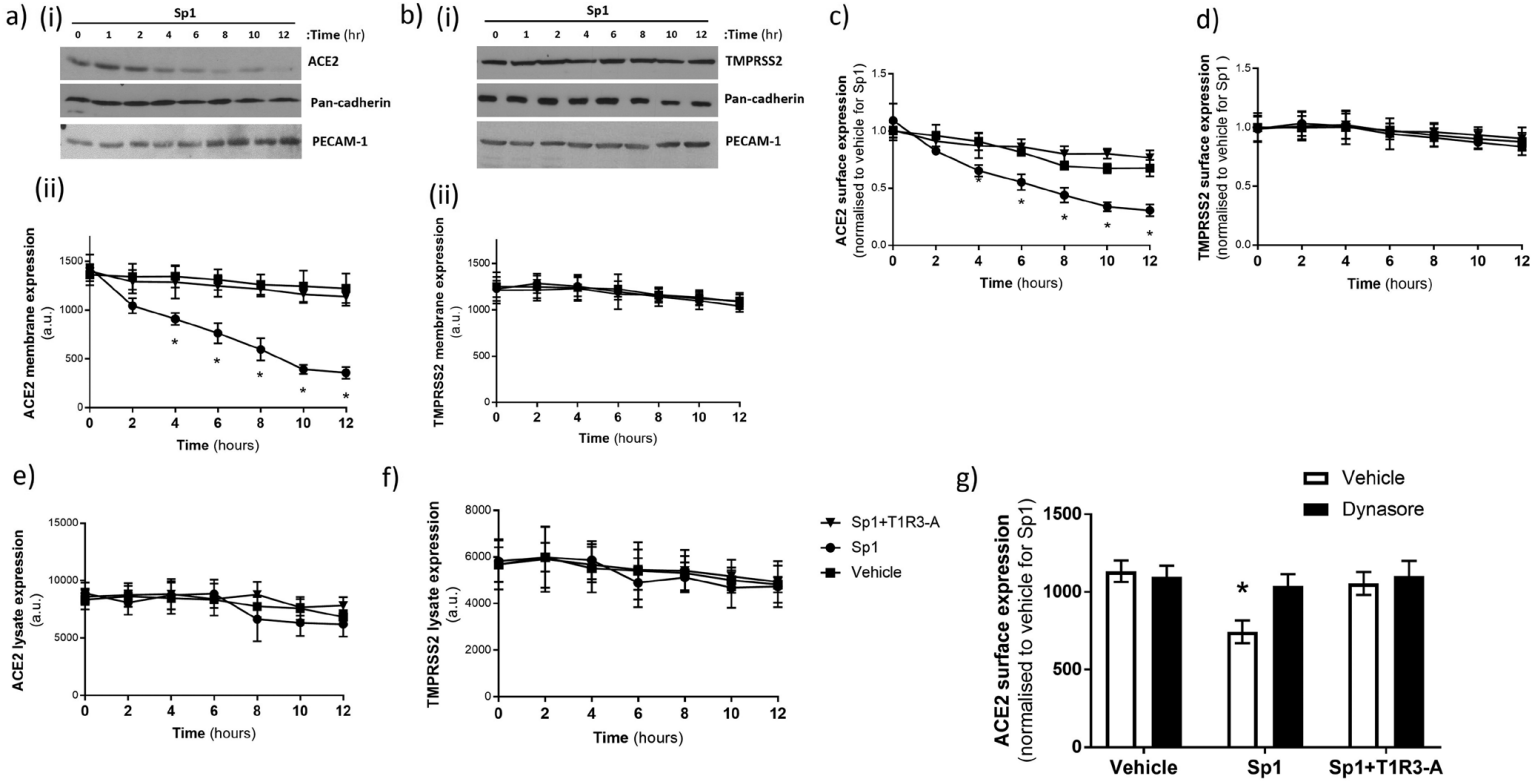
T1R3‐A attenuates Sp1‐induced internalisation of ACE2 at the pulmonary endothelial cell surface through intracellular trafficking. HLMVEC were treated with Sp1 (10 nM) for up to 12 h, or vehicle (media), with T1R3‐A (sucralose, 100 μM) or vehicle for sucralose (H_2_O). For panel (d), Dynasore was added to cell treatment as an inhibitor of trafficking. ACE2 (a, c, e and g) and TMPRSS2 (b, d, f) expression was assessed with Western blotting (a, b, e, f) of endothelial cell membrane preparations (a and b) and whole lysate (e and f) and by whole cell ELISA (c, d, g). For Western blots, a representative blot (i) and quantification (ii) are included. Data are presented as mean ± SEM. *n* = 5–6. *p < 0.05 versus vehicle for Sp1.

Studies indicate that SARS‐CoV‐2 cannot replicate in endothelial cells; however, we (Figure 1) and others [28, 29] have demonstrated that Sp1 is sufficient to cause microvascular cell injury. It is therefore likely that endothelial cell damage is associated with ACE2‐Sp1 interaction and the resulting intracellular signalling. Therefore, our next experiments investigated whether T1R3‐A treatment impacts ACE2 expression in HPMVEC in the presence and absence of Sp1. We first studied the effect of Sp1 on ACE2 and protein transmembrane protease serine Type 2 (TMPRSS2), which also play a key role in SARS‐CoV‐2 infection [13] at the endothelial cell surface by isolating plasma membrane preparations from cells exposed to Sp1 for different time points. Isolation was confirmed by PECAM expression (Figure 3a,b). From 4 h onwards, Sp1 induced a significant reduction of ACE2 expression at the endothelial cell surface which was not observed in HPMVEC exposed to T1R3‐A and Sp1 together (Figure 3a). In contrast, TMPRSS2 expression at the cell surface was not changed by Sp1 exposure in the presence or absence of T1R3‐A (Figure 3b). These findings were mirrored with whole cell ELISA analysis of ACE2 (Figure 3c,d). Interestingly, analysis of protein expression in total cell lysate showed no change in ACE2 (Figure 3e) or TMPRSS2 (Figure 3f) expression levels. These findings suggest that T1R3‐A blocks Sp1‐induced internalisation of ACE2; therefore, we further studied this using the cell‐permeable dynamin inhibitor, Dynasore. As would be expected, Sp1‐mediated reduction in ACE2 cell surface expression was blocked by Dynasore treatment; however, there was no effect of the dynamin inhibitor in settings of Sp1 and T1R3‐A exposure (Figure 3g). These findings support the notion that T1R3‐A exposure blocks ACE2 internalisation, maintaining expression at the cell surface.

### 3.4 T1R3‐A Maintains ACE2 Expression at the Endothelial Cell Surface by Promoting T1R3‐ACE2 Interaction

**FIGURE 4:**
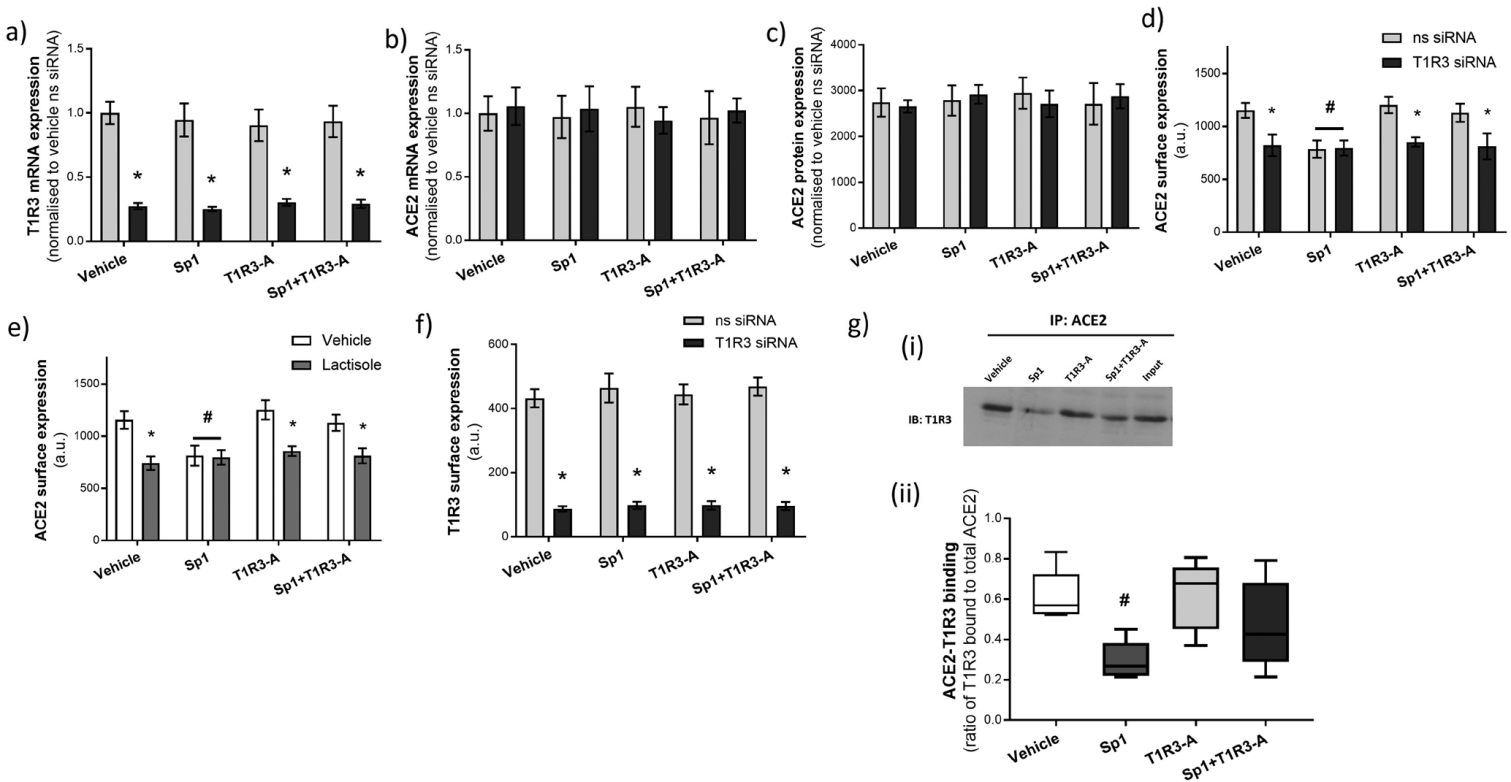
T1R3‐A maintains ACE2 expression at the endothelial cell surface by promoting T1R3‐ACE2 interaction. Panels a–e: HLMVEC were exposed to siRNA specific to T1R3, or a non‐specific (ns) scrambled control, for 44 h prior to treatment with Sp1 (10 nM) for 4 h, or vehicle (media), with T1R3‐A (sucralose, 100 μM) or vehicle for sucralose (H_2_O) in the presence or absence of the T1R3 inhibitor, lactisole (3 μM) (e). mRNA expression of TAS1R3 (a) and ACE2 (b) was measured using RT‐PCR. ACE2 protein expression in whole lysate (c) and cell surface (d and e) was assessed using Western blotting and whole cell ELISA respectively. T1R3 cell surface expression was measured with whole cell ELISA (f). Immunocomplexes from ACE2 were probed with antibodies specific to T1R3 (g) with representative blot (i) and quantification (ii) included. Data are presented as mean ± SEM. *n* = 6–7. *p < 0.05 versus ns siRNA vehicle; ^#^ *p* < 0.05 versus vehicle for Sp1.

The next studies sought to understand the effect of T1R3‐A in regulating Sp1‐mediated injury of the pulmonary endothelial monolayer. To investigate whether T1R3‐A protects against Sp1‐induced barrier disruption through a T1R3‐dependent pathway, experiments were conducted in HPMVEC following molecular inhibition of the GPCR. siRNA knockdown of T1R3 resulted in a 72.56% ± 0.66% decrease in T1R3 mRNA expression which was maintained following exposure to Sp1, T1R3‐A and Sp1 with T1R3‐A (Figure 4a). T1R3 knockdown of expression had no impact on ACE2 mRNA (Figure 4b) or total cell lysate protein expression (Figure 4c). However, ACE2 cell surface expression was significantly decreased in HPMVEC with T1R3 knockdown following vehicle or T1R3‐A treatment (Figure 4d). In both non‐specific and T1R3‐specific siRNA treatment, cells exposed to Sp1 displayed a significant decrease in ACE2 cell surface expression (Figure 4d). Interestingly, ACE2 surface expression in T1R3 knockdown cells was significantly decreased in the presence of both Sp1 and T1R3‐A (Figure 4d). In the presence of the sweet taste inhibitor, lactisole, ACE2 surface expression was similarly decreased in the presence of both Sp1 and T1R3‐A (Figure 4e). These changes were accompanied with a significant decrease in T1R3 cell surface expression in T1R3 knockdown cells, but no effect of specific treatment with either T1R3‐A or Sp1 (Figure 4f). Taken together, these findings indicate that T1R3 is needed to maintain ACE2 expression at the endothelial cell surface in vehicle‐treated cells. Furthermore, in the absence of T1R3 expression, T1R3‐A is not able to attenuate Sp1‐induced ACE2 internalisation from the endothelial cell surface. A previous study has indicated a potential interaction between T1R3 and ACE2 in human fungiform taste cells [21] therefore, to investigate how T1R3 maintains ACE2 expression at the cell surface, we next established whether ACE2‐T1R3 binding occurred in the pulmonary endothelium in vitro. Immunoprecipitation studies demonstrate expression of T1R3 in ACE2 immunocomplexes which was significantly reduced following Sp1 exposure (Figure 4g). Sp1‐mediated attenuation of ACE2‐T1R3 interaction was alleviated with T1R3‐A exposure (Figure 4g). To further understand the effect of T1R3 in regulating endothelial barrier function, our final studies assessed Sp1‐mediated barrier dysfunction in cells following T1R3 siRNA knockdown. In cells treated with either non‐specific or T1R3‐specific siRNA, Sp1 exposure resulted in a significant increase in gap formation and FITC‐dextran monolayer flux, alongside reduced TEER, VE‐cadherin surface expression and accumulation of ROS (Table 1). Interestingly, T1R3 knockdown blocked the protective effect exerted by T1R3‐A on Sp1‐induced barrier disruption and oxidative stress (Table 1).

**TABLE 1:** Molecular inhibition of T1R3, using specific siRNA, blocks the protection which T1R3‐A exerts on Sp1‐mediated barrier disruption and oxidative stress.

	Non‐specific siRNA			T1R3‐specific siRNA		
	Vehicle	Sp1	Sp1 + T1R3‐A	Vehicle	Sp1	Sp1 + T1R3‐A
Gap formation (%)	100 ± 5.5	180.3 ± 16.3*	103.1 ± 18.4	102.5 ± 11.8	180.9 ± 11.8*	181.53 ± 15.0*, ^#^
Transwell fluorescence (r.f.u.)	865 ± 71	2342 ± 258*	860 ± 117	855 ± 81	2421 ± 384*	2397 ± 203*, ^#^
Trans‐endothelial electrical resistance (ohms)	1 ± 0.033	0.653 ± 0.026*	0.958 ± 0.030	0.991 ± 0.028	0.633 ± 0.026*	0.673 ± 0.039*, ^#^
VE‐cadherin surface expression (r.f.u. ×100)	48.91 ± 3.37	26.65 ± 2.03*	47.32 ± 4.65	50.34 ± 2.18	35.28 ± 2.63*	31.03 ± 1.56*, ^#^
ROS (%)	100 ± 14.4	218.6 ± 22.8*	139.9 ± 5.27	111.1 ± 13.5	211.1 ± 16.6*	217.7 ± 14.5*, ^#^

*Note:* Data are presented as mean ± SEM. *n* = 6–7.

* *p* < 0.05 versus vehicle‐treated cells.

^#^ *p* < 0.05 versus non‐specific siRNA.

Taken together, these data indicate that T1R3 interacts with ACE2 which potentially enhances ACE2 expression at the endothelial cell surface. We propose that T1R3‐A blocks Sp1‐induced ACE2 internalisation to protect the pulmonary endothelium against barrier disruption in vitro.

## 4 Discussion

In this study, we demonstrate the significant activation of lung endothelial cells with breakdown of the lung microvasculature in vitro following exposure to the Sp1 peptide from the SARS‐CoV‐2 virus. We observe that exposure to an agonist for T1R3 (T1R3‐A) blocks this barrier disruption but not the inflammation in a T1R3‐specific manner. We further show that T1R3‐A attenuates ACE2 internalisation by promoting T1R3 to anchor ACE2 to the endothelial cell surface. We therefore propose that agonists for T1R3 offer a novel therapeutic approach to protect against SARS‐CoV‐2‐induced barrier disruption and have the potential to reduce the severe hypoxaemia seen in patients with COVID‐19 related ARDS.

Our findings demonstrate that exposure to the SARS‐CoV‐2 spike protein S1 subunit (Sp1) is sufficient to induce key features of endothelial cell activation in the lung microvasculature in a dose‐dependent manner. Increase in gap formation and monolayer permeability along with reduced VE‐cadherin and increased ICAM‐1 cell surface expression, and elevated VE‐cadherin tyrosine phosphorylation, following 24‐h exposure to Sp1, demonstrate significant breakdown of the pulmonary endothelium. This confirms previous findings by Colunga Biancatelli et al. [12] where HSP‐dependent increase in trans‐endothelial resistance was noted in human lung microvascular endothelial cells and by Perico et al. [30], where elevated ICAM‐1 expression and von Willebrand depiction was noted in human microvascular endothelial cells following Sp1 exposure. Further, in vivo studies have also been demonstrated to increase vascular permeability with intratracheal administration of Sp1 in mice significantly increasing protein concentration, monocytes and neutrophils in bronchoalveolar fluid (BALF) [12]. In addition to breakdown of the endothelial monolayer, oxidative stress and elevated TGF‐β, IL‐8, and IL‐6 release were observed following Sp1 exposure in our studies. These studies agree with findings from the in vivo Sp1 model studied by Colunga Biancatelli et al. [12] and are indicative of the ‘cytokine storm’ which is a feature of endothelialitis in patients with COVID‐19 [9]. Indeed, in vitro studies with human microvascular endothelial cells show that Sp1 activates the complement system, with heightened C3 and C5‐b9 deposition on endothelial cells, to enhance platelet aggregate formation and mimic the thrombosis seen in COVID‐19 patients [30, 31, 32]. Interestingly, systemic instillation of Sp1 in mice induces a tissue injury with alveolar thickening similar to that seen with LPS‐induced mouse models of ARDS [11, 33]. The SARS‐CoV‐2 spike protein used in the present studies is produced in *E. coli* ; therefore, it is possible that the inflammatory and barrier disruptive effects of the peptide are linked to an endotoxin, rather than Sp1 peptide‐specific, effect. Lubow and Weindl [34] recently demonstrated that the spike protein from SARS‐CoV‐2 activated TLR4 signalling in THP‐1 differentiated macrophages confirming a pro‐inflammatory signalling cascade from bacterial‐derived Sp1 which is typical of ARDS. Whilst there is controversy in the field with regards to the clinical similarity between ‘classical’ ARDS and ARDS in COVID‐19 patients [35], it is widely accepted that endothelial activation, and the resulting signalling which occurs in both forms of ARDS represents a therapeutic target. For example, single‐cell RNA sequencing shows that the TLR4 pathway, a well‐established ‘classical’ ARDS target, is crucial in the endothelial response to SARS‐CoV‐2 [36, 37]. Therefore, therapeutic targets which have been identified to improve endothelial function in ‘classical’ ARDS studies have significant potential to act on the endothelium in settings of COVID‐19.

In the present study, we focused on the novel GPCR, T1R3, as our previous work has identified that T1R3 agonists exert vascular protective effects in vitro and in vivo in both VEGF‐ and LPS‐induced models of endothelial cell activation [18, 24, 38]. T1R3 functions as a sweet taste sensor in taste organs, either as a heterodimer with T1R2 or as a homodimer, and is stimulated by high concentrations of glucose (> 300 mM) or low concentrations of artificial sweeteners (< 1 mM) [39, 40]. In the present study, we used sucralose as a T1R3 agonist however there are a range of potential agonists, such as acesulfame K and aspartame, which similar binding affinity which would be of interest to investigate for similar protective effects [41]. We have previously identified T1R3 expression in the pulmonary microvasculature and noted that the agonist, T1R3‐A, significantly protects against pulmonary vascular leak using an LPS model of ARDS in vitro and a bacterial model in vivo [18]. Similarly, in the present study, we observed that T1R3‐A abrogated Sp1‐induced barrier disruption and VE‐cadherin expression at the cell surface. Interestingly, T1R3‐A had no effect on Sp1‐induced cytokine release (TGF‐β, IL‐8, IL‐6) or ICAM‐1 expression. These findings differ from those in macrophages, where trehalose sugar was shown to attenuate macrophage IL‐1β expression through T1R3 [42], however this may represent a key difference between disaccharides and artificial sweeteners, as well as the different inflammatory responses in endothelial cells versus macrophages. Our findings suggest a specific protective mechanism of T1R3 in maintaining the barrier integrity of the lung microvasculature. Indeed, we have previously demonstrated that T1R3‐A attenuates LPS‐induced phosphorylation of the proteins PAK, MLC2 and Src which are important for adherens junctions and actin remodelling [18, 43]. Previous studies have indicated that breakdown of the lung endothelium caused by Sp1 is linked to elevated Hsp90 resulting in Akt phosphorylation and reduced cofilin phosphorylation [44]. We have previously demonstrated that T1R3‐A does not affect Hsp90 expression or LPS‐induced phosphorylation of cofilin [18] but, interestingly, in the retinal microvasculature, T1R3‐A attenuates VEGF‐induced angiogenic processes through blocking Akt phosphorylation [38]. It is therefore possible that, in the pulmonary microvasculature, T1R3‐A blocks Sp1‐induced Akt phosphorylation to exert a barrier protective effect. Indeed, Akt inhibitors have been indicated to decrease lung damage in patients with COVID‐19; however, this is likely linked to an anti‐inflammatory effect and downregulation of ACE2 expression [45]. Furthermore, there are a wide range of other potential therapeutic signalling pathways which T1R3 could be regulating to exert this barrier‐protective effect on the lung endothelium. For example, SARS‐CoV‐2 RBD has been shown to inactivate ENaC, resulting in oxidative stress and tissue factor generation linked to barrier disruption [46]. Therefore, it is possible that T1R3 could impact ENaC activation. Interestingly, ENaC can act as a taste sensor and has been identified in the same cells and tissue as T1R3 [47, 48], suggesting a potential link between the taste receptor and sodium channel. Thus, there is a need for further study into the range of signalling and regulation of ACE2 to understand how T1R3‐A could be protecting the pulmonary endothelium in vascular leak models of COVID‐19.

It is well‐understood that Sp1 on SARS‐CoV‐2 binds to the ACE2 receptor on the endothelial cell surface through the RBD domain, to promote internalisation of the Sp1:ACE2 complex via endocytosis [13, 49, 50]. In further investigation of Sp1‐induced signalling in the endothelial cell, we identified the direct effect of T1R3‐A on ACE2 expression at the endothelial cell surface. Whilst Sp1 has no effect on TMPRSS2 protein expression or ACE2 protein or mRNA expression in the cell, it significantly reduced ACE2 surface expression. This is not surprising as TMPRSS2 is responsible for cleaving the Sp1 from the whole SARS‐CoV‐2 virus, a role which is not needed where Sp1 is directly administered to cells [10, 51]. T1R3‐A‐mediated maintenance of ACE2 expression at the endothelial cell surface, in the presence of Sp1, has not been previously shown and indicates a mechanism through which the T1R3 agonist blocks ACE2:Sp1 binding to inhibit endocytosis into the endothelial cell, and therefore block the subsequent barrier disruption which occurs. It is interesting that molecular inhibition of T1R3 significantly blunts basal ACE2 expression but has no further impact on Sp1‐induced loss of ACE2 surface expression. This may be due to a sensitivity limitation with our assay. That is, with Sp1 treatment and with any exposure to T1R3 siRNA, irrespective of the co‐treatment, the surface expression levels of ACE2 reached a similar level suggesting a lower limit level of the assay. Alternatively, in endothelial cells, ACE2 expression may not decrease below this lower level to maintain functional capacity; however, given mRNA expression is unaffected, this would need to be further investigated. Whilst our studies demonstrate the mRNA and protein expression of ACE2, as well as membrane localisation of the protein, there is controversy in the field regarding ACE2 in the endothelium. In humanised ACE2 transgenic mice, Muhl et al. [52] noted no ACE2 expression in endothelial cells as opposed to pericytes which show high expression. Similarly, in vitro transcriptomics by McCracken et al. [53] indicate low or no basal ACE2 expression as compared to epithelial cells. In contrast, several studies demonstrate the localisation and role of ACE2 in microvascular endothelial cells [10, 15, 54, 55, 56]. Whilst our studies demonstrate ACE2 presence in the lung endothelium, it is worth noting that our study used cultured primary human lung microvascular endothelial cells rather than whole tissue; therefore, further study is needed to show the in vivo value of our findings. Whilst T1R3‐A did not affect T1R3 expression at the endothelial cell surface, the agonist significantly increased the ACE2‐T1R3 interaction. Previous studies have demonstrated co‐expression of T1R3 with ACE1 in the fungiform and circumvallate papillae of taste bud cells and interaction between T1R3 and ACE2 in human fungiform taste cells [19, 21]. This is the first study to demonstrate ACE2:T1R3 binding in the vasculature and to identify a novel approach to regulate this interaction. It is worth noting that immunoprecipitation studies in the present work used whole cell lysate; therefore, they could capture ACE2:T1R3 binding at any cellular localisation. Given there is no change in ACE2 expression in whole lysates, it is likely that the ACE2:T1R3 interaction is occurring at the endothelial cell surface; however, further studies, specifically immunofluorescence co‐localisation studies, are needed to fully investigate this association including the potential interaction sites in both receptors.

These studies are the first to demonstrate a protective effect of T1R3 activation on SARS‐CoV‐2‐associated endothelial cell damage by anchoring ACE2 to the endothelial cell surface and blocking internalisation. These findings offer a new therapeutic target, T1R3, in the treatment of endothelial cell damage and lung injury in COVID‐19. However, it is worth noting that studies have been performed in human cells rather than in vivo and the therapeutic approach, T1R3‐A, was administered to cells at the same time as the injury peptide, Sp1. Therefore, further studies are needed to understand whether T1R3‐A can rescue Sp1‐induced barrier disruption in the microvasculature. There is, at present, no literature which links artificial sweetener consumption and COVID‐19; therefore, it is difficult to assess whether T1R3‐A in the diet has an impact on vascular injury. However, it is well‐established that artificial sweeteners cause gut dysbiosis and increase pathogenic markers in bacteria from the microbiota [57, 58, 59]. Furthermore, in vivo studies demonstrate that T1R3‐A consumption limits T cell differentiation and proliferation with a dampened response to models of T cell‐associated autoimmunity [60]. Therefore, it is unlikely that a simple change in sweetener consumption would be a recommended therapeutic approach in targeting endothelial injury in COVID‐19.

## Author Contributions

**Zsuzsanna Kertesz:** conceptualization (equal), investigation (equal), methodology (equal), writing – review and editing (equal). **Lewis Spurrier‐Best:** investigation (equal), writing – review and editing (equal). **Havovi Chichger:** conceptualization (equal), data curation (lead), formal analysis (lead), investigation (equal), methodology (equal), project administration (lead), resources (lead), writing – original draft (lead).

## Funding

Research England Quality‐Related (QR) Research funding awarded to H.C. by the Faculty of Science and Engineering, Anglia Ruskin University.

## Conflicts of Interest

The authors declare no conflicts of interest.

## Data Availability

All data collected from this study will be available by request to the corresponding author.
